# Chemokines and Growth Factors Produced by Lymphocytes in the Incompetent Great Saphenous Vein

**DOI:** 10.1155/2019/7057303

**Published:** 2019-01-10

**Authors:** Ewa Grudzińska, Sławomir Grzegorczyn, Zenon P. Czuba

**Affiliations:** ^1^School of Medicine with the Division of Dentistry in Zabrze, Medical University of Silesia in Katowice, Department of Microbiology and Immunology, Jordana 19, 41-808 Zabrze, Poland; ^2^School of Medicine with the Division of Dentistry in Zabrze, Medical University of Silesia in Katowice, Department of Biophysics, Jordana 19, 41-808 Zabrze, Poland

## Abstract

The role of cytokines in the pathogenesis of chronic venous disease (CVD) remains obscure. It has been postulated that oscillatory flow present in incompetent veins causes proinflammatory changes. Our earlier study confirmed this hypothesis. This study is aimed at assessing chemokines and growth factors (GFs) released by lymphocytes in patients with great saphenous vein (GSV) incompetence. In 34 patients exhibiting reflux in GSV, blood was derived from the cubital vein and from the incompetent saphenofemoral junction. In 12 healthy controls, blood was derived from the cubital vein. Lymphocyte culture with and without stimulation by phytohemagglutinin (PHA) was performed. Eotaxin, interleukin 8 (IL-8), macrophage inflammatory protein 1 A and 1B (MIP-1A and MIP-1B), interferon gamma-induced protein (IP-10), monocyte chemoattractant protein-1 (MCP-1), interleukin 5 (IL-5), fibroblast growth factor (FGF), granulocyte colony-stimulating factor (G-CSF), granulocyte-macrophage colony-stimulating factor (GM-CSF), platelet-derived growth factor-BB (PDGF-BB), and vascular endothelial growth factor (VEGF) were assessed in culture supernatants by a Bio-Plex assay. Higher concentrations of eotaxin and G-CSF were revealed in the incompetent GSV, compared with the concentrations in the patients' upper limbs. The concentrations of MIP-1A and MIP-1B were higher in the CVD group while the concentration of VEGF was lower. In the stimulated cultures, the concentration of G-CSF proved higher in the incompetent GSV, as compared with the patients' upper limbs. Between the groups, the concentration of eotaxin was higher in the CVD group, while the IL-5 and MCP-1 concentrations were lower. IL-8, IP-10, FGF, GM-CSF, and PDGF-BB did not reveal any significant differences in concentrations between the samples. These observations suggest that the concentrations of chemokines and GFs are different in the blood of CVD patients. The oscillatory flow present in incompetent veins may play a role in these changes. However, the role of cytokines in CVD requires further study.

## 1. Introduction

Chronic venous disease (CVD) affects up to 85% of the population, and more advanced clinical changes (C3–C6 in the Clinical-Etiology-Anatomy-Pathophysiology (CEAP) classification) occur in about 30% of the population [1–4]. Its exact pathogenesis remains unclear. However, the impact of inflammatory processes is considered crucial for venous wall remodeling [5–11]. Reflux in the incompetent veins causes oscillatory flow, with blood moving towards the heart during the contraction of the muscular pump of the calf and backwards during the relaxation of the calf [12]. It has been demonstrated that these flow changes cause the release of proinflammatory cytokines by endothelial cells and lead to leukocyte-mediated inflammatory reactions [12–16]. Our recent publication demonstrated that proinflammatory cytokines are released by lymphocytes in higher concentrations in the incompetent veins [17].

In order to further investigate the role of cytokines released by lymphocytes in CVD, we studied two other panels: chemotactic cytokines and growth factors (GFs). Both these cytokine groups have previously been described as released in higher concentrations by endothelium subject to hypoxia [18]. Following our previous findings which confirmed the proinflammatory state in CVD, it seemed probable that the levels of chemotactic cytokines should be elevated in the incompetent vein, recruiting leukocytes and promoting the inflammatory process. The influence of GFs on the histological changes in CVD also seemed possible. The incompetent vein wall is known to be distorted, with a degraded extracellular matrix [19], damaged intima [20–23], and disorganized, hypertrophic media [24, 25]. An imbalance between collagen and elastin has been observed, with lower content of elastin and collagen type III and higher content of collagen type I [26–29]. These changes are linked to a higher metalloproteinase activity [19, 30], dysregulated apoptosis [14, 24, 31], and elevated smooth muscle proliferation [25]. Increased numbers of vasa vasorum in varicose veins have also been observed [23]. The GFs have been demonstrated to play a role in regulating the metalloproteinase activity [32]. Moreover, they take part in neovascularisation. Therefore, their role in CVD progression seems possible.

Few papers describe cytokines in CVD [9], and there are no studies concerning the role of chemokines and GFs released by lymphocytes in this disease. No differences in the lymphocyte percentage were observed in the varicose veins when compared to healthy veins [6, 33], and the lymphocytes were shown to have an important role in venous ulcer development [34].

We expected to find different cytokine production in the incompetent vein with oscillatory flow when compared to the same patients' healthy cubital vein with laminar (unidirectional) flow. The circulating lymphocytes are also subject to contact with turbulent flow and pathologically changed endothelium of the incompetent vein; therefore, differences in cytokine concentrations in the cubital blood from healthy subjects and CVD patients were expected. Finally, the lymphocytes in CVD group may react differently to stimulating agents than the lymphocytes in the healthy group.

## 2. Materials and Methods

The study has been carried out in accordance with the Declaration of Helsinki and approved by the Bioethical Committee of the Medical University of Silesia (KNW/0022/KB1/31/I/12). All participants gave their written informed consent for the study.

The CVD group consisted of 34 primary CVD patients with great saphenous vein (GSV) incompetence confirmed by the Doppler ultrasound examination. The reflux at saphenofemoral junction (reflux time > 0.5 s) was confirmed in all patients in standing position, with blood flow induced by manual squeezing. The control group included 12 volunteers with healthy GSV confirmed by the Doppler ultrasound. The exclusion criteria involved history of venous thrombosis, pregnancy, diabetes, any inflammatory diseases present in the past two weeks, alcohol abuse, smoking, ulceration on the examined limb during the last month, and intake of anti-inflammatory drugs within the past two weeks.

Blood samples were obtained from the cubital vein in both groups, collected to vials containing heparin (10 IU/ml of blood). Consequently, patients from the CVD group underwent standard surgical procedure of GSV stripping, with femoral nerve block and additional local anaesthesia. The inguinal incision and visualization of the GSV were performed. A blood sample from the GSV directly below the incompetent saphenofemoral junction was collected into a heparinized vial. All samples were immediately transferred to the laboratory, and the temperature of 37°C was maintained. Cultures of lymphocytes were prepared either with lymphocyte-stimulating phytohemagglutinin (PHA) or with a medium as follows:

The lymphocytes were separated by the use of Histopaque gradients (1.119 g/ml and 1.077 g/ml). After centrifugation (700 × *g*, 30 min), the separated lymphocytes were transferred to another vial and washed twice with phosphate-buffered saline (PBS) (250 × *g*, 10 min). Microscopic morphological assessment of cell population was performed, and no differences were found between the groups. No significant contamination by other cells was found in the samples.

A suspension of 2 MM lymphocyte cells/ml of medium (Roswell Park Memorial Institute (RPMI) 1640, 10% bovine serum, penicillin 100 U/ml, and streptomycin 100 *μ*g/ml) was prepared. 0.5 ml of this suspension was added to a 0.5 ml of PHA solution (20 *μ*g PHA/ml of medium) and for no-stimulation samples, 0.5 ml of the suspension to a 0.5 ml of medium. These suspensions were incubated for 24 h in 37°C, 5% CO_2_ atmosphere, and 99% humidity. After incubation and centrifugation (250 × *g*, 10 min), the supernatant was collected into the Eppendorf vials and stored at -80°C.

Assessed panels included chemotactic factors: eotaxin, interleukin 8 (IL-8), macrophage inflammatory protein 1 A and 1B (MIP-1A and MIP-1B), interferon gamma-induced protein (IP-10), monocyte chemoattractant protein-1 (MCP-1), and GFs: interleukin 5 (IL-5), fibroblast growth factor (FGF), granulocyte colony-stimulating factor (G-CSF), granulocyte-macrophage colony-stimulating factor (GM-CSF), platelet-derived growth factor-BB (PDGF-BB), and vascular endothelial growth factor (VEGF).

The samples were thawed directly before the Bio-Plex assay. The assay uses magnetic beads with anticytokine immunoglobulins to assess simultaneously the concentrations of many cytokines. The samples were processed following the manufacturer's instructions (Bio-Plex Pro™ Human Cytokine Assays, Bio-Rad Laboratories) and read using Bio-Rad Bio-Plex™ 200 System with Bio-Plex Manager™ Software. The statistical analysis was performed with the use of STATISTICA 10.0 software. The cytokine data were not normally distributed; therefore, nonparametric tests were applied. Mean/median differences were analyzed by Student's paired *t-*test, the Wilcoxon signed-rank test, or the Mann-Whitney *U* test. The leukocyte count and lymphocyte percentage had normal distribution; therefore, Student's *t*-test was applied.

## 3. Results and Discussion

### 3.1. Results

The CVD group consisted of 34 patients, 85% of which were women. Median age was 47 ± 25 (21-68). The patients belonged to clinical CEAP classes C2-C3, with 43% in C2 and 57% in C3. The control group consisted of 12 patients, 92% of which were women. Median age was 36 ± 27 (29-64). The white blood cell count was mean 5.6 × 10^3^/*μ*l (3.7-8.8 × 10^3^/*μ*l) in the CVD group and 5.9 × 10^3^/*μ*l (4.6-7.5 × 10^3^/*μ*l) in the control group. The lymphocyte percentage was mean 39% (22%-47%) in the CVD group and 36% (23%-45%) in the control group. There were no statistically significant differences between the groups.

In the samples cultured without stimulation, significantly higher concentrations of eotaxin and G-CSF were found in the incompetent GSV samples in comparison with the cubital vein samples of the same patients (results are expressed as median ± quartile deviation and range).

**Figure 1:**
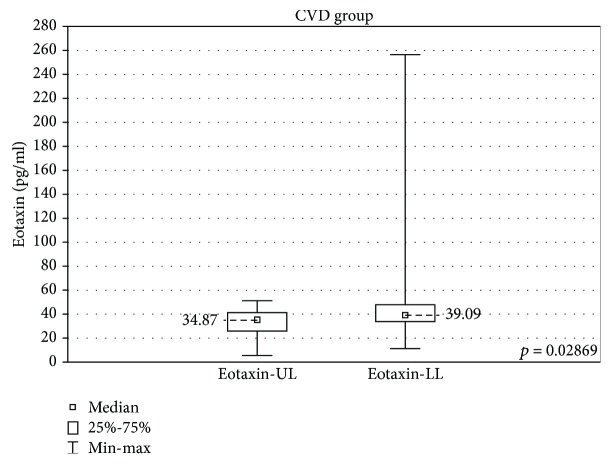
Comparison of eotaxin concentrations in the upper (eotaxin-UL) and lower limb samples (eotaxin-LL) in the CVD groups, cultured without stimulation.

**Figure 2:**
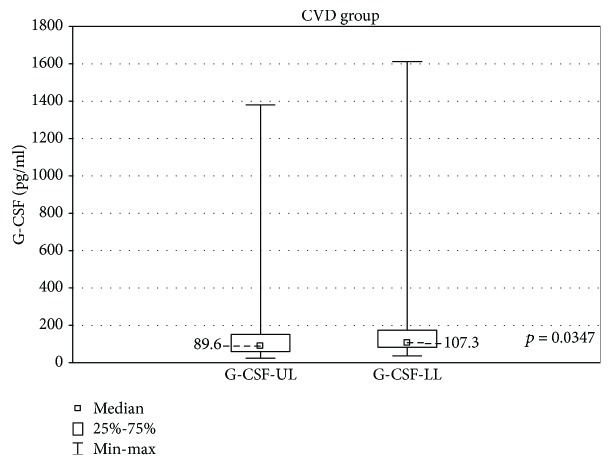
Comparison of G-CSF concentrations in the upper (G-CSF-UL) and lower limb samples (G-CSF-LL) in the CVD groups, cultured without stimulation.

Eotaxin: 39.09 ± 14.1 (11.4-256.8) pg/ml vs 34.87 ± 15.47 (5.6-51.34) pg/ml, *p* < 0.05 and G-CSF: 107.4 ± 91.5 (36.3-1613) pg/ml vs 89.6 ± 91.9 (24.7-1381) pg/ml, *p* < 0.05. The above results are presented in Figures 1 and 2.

**Figure 3:**
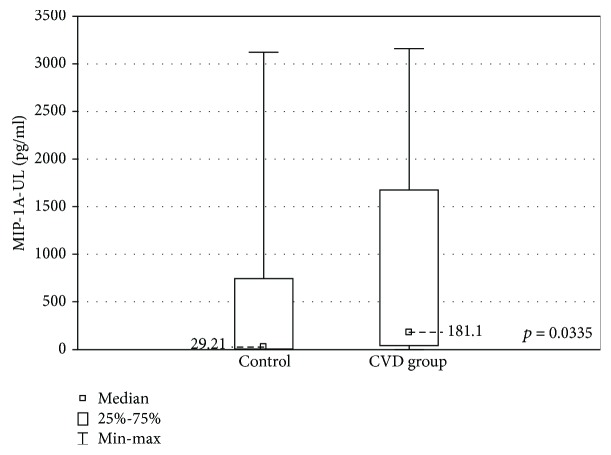
Comparison of the MIP-1A concentrations in the upper limb samples (MIP-1A-UL) between the CVD and control groups, cultured without stimulation.

**Figure 4:**
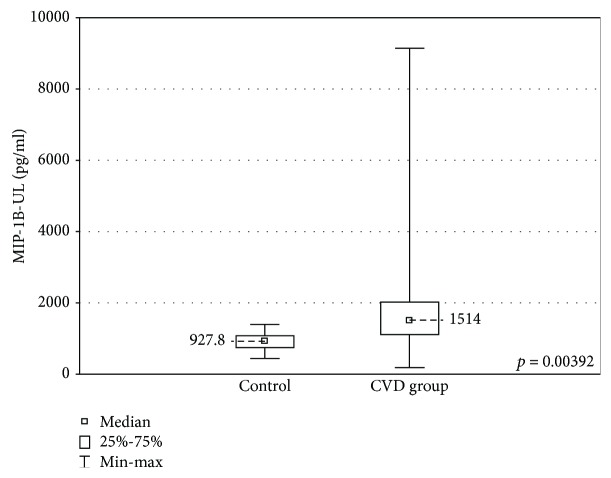
Comparison of the MIP-1B concentrations in the upper limb samples (MIP-1B-UL) between the CVD and control groups, cultured without stimulation.

**Figure 5:**
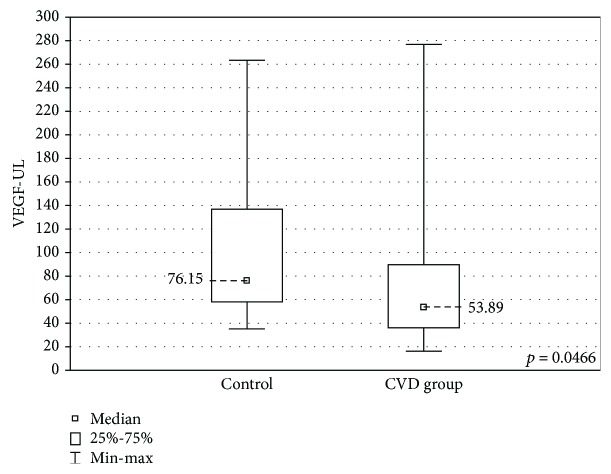
Comparison of the VEGF concentrations in the upper limb samples (VEGF-UL) between the CVD and control groups, cultured without stimulation.

When the upper limb samples cultured without stimulation were compared between the groups, significantly higher concentrations of MIP-1A and MIP-1B were found in the upper limb samples of the CVD group (MIP-1A: 181.1 ± 1633 (2.18-3163) pg/ml vs 29.2 ± 3123 (2.7-3125) pg/ml, *p* < 0.05 and MIP-1B: 1514 ± 905.1 (185.6-9142) pg/ml vs 927.8 ± 325.1 (444.3-1396) pg/ml, *p* < 0.01). The CVD group showed lower concentrations of VEGF (53.9 ± 53.3 (17.4-276.8) pg/ml vs 76.2 ± 78.6 (35.3-263.5) pg/ml, *p* < 0.05). These results are presented in Figures 3–5.

PHA did not cause significant changes in the concentrations of MIP-1B and PDGF-BB in any group. IL-8 and VEGF did not show any difference in concentrations in the control group. PHA did not cause significant changes in the IL-5 concentrations in the CVD group. FGF did not show any significant changes in the concentrations in the PHA cultures of the lower limb samples in the CVD group. The GM-CSF concentrations were higher in the PHA cultures only in the upper limb samples of the CVD group. The remaining PHA-stimulated samples had significantly higher cytokine concentrations than the unstimulated samples (Table 1).

The magnitude of lymphocyte stimulation by PHA was analyzed and no statistically significant differences were found. The exception is MCP-1 which showed a more significant increase in the concentration after PHA stimulation in the control group, as compared with the examined group (median increase 899 ± 1391 ((-2302)-2681) pg/ml vs 548 ± 414 ((-1341)-2072) pg/ml).

**Figure 6:**
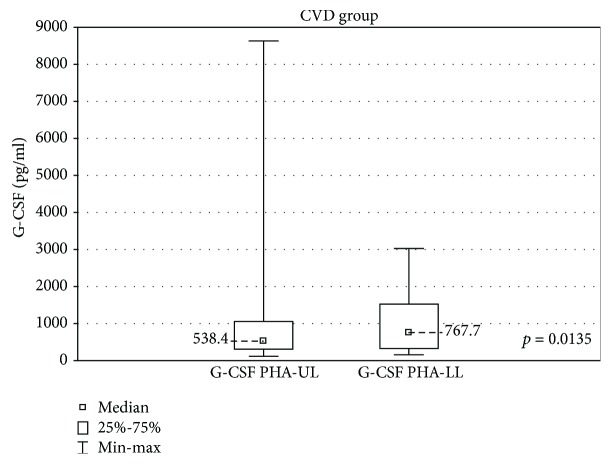
Comparison of G-CSF concentrations in the upper (G-CSF PHA-UL) and lower limb samples (G-CSF PHA-LL) in the CVD group, cultured with PHA stimulation.

In the samples cultured with stimulation, in the CVD group, the GSV samples had a significantly higher G-CSF concentration as compared with the upper limb samples (767.7 ± 1197 (160.2-3030) pg/ml vs 538.4 ± 747.3 (115.7-8630) pg/ml, *p* < 0.05) (Figure 6).

**Figure 7:**
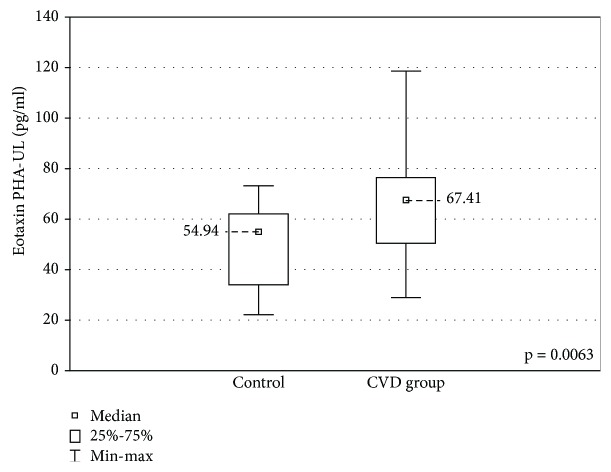
Comparison of the eotaxin concentrations in the upper limb samples (eotaxin PHA-UL) between the CVD and control groups, cultured with PHA stimulation.

**Figure 8:**
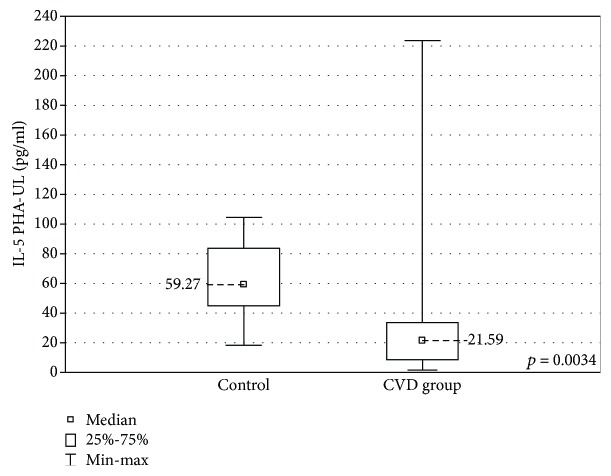
Comparison of the IL-5 concentrations in the upper limb samples (IL-5 PHA-UL) between the CVD and control groups, cultured with PHA stimulation.

**Figure 9:**
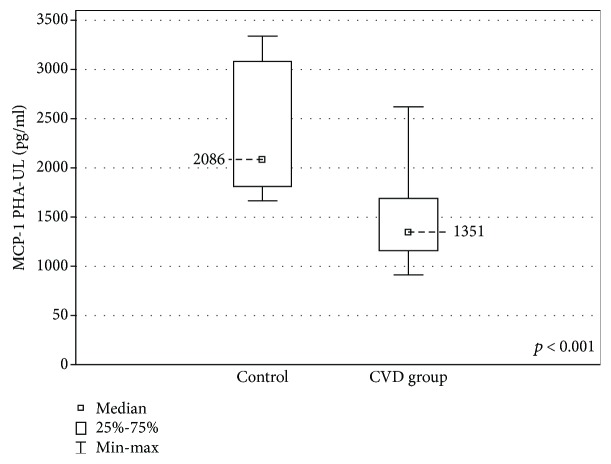
Comparison of the MCP-1 concentrations in the upper limb samples (MCP-1 PHA-UL) between the CVD and control groups, cultured with PHA stimulation.

When the upper limb samples cultured with stimulation were compared between the groups, a higher concentration of eotaxin was found in the CVD group (67.41 ± 25.9 (29.0-118.7) pg/ml vs 54.9 ± 28.0 (22.15-73.25) pg/ml, *p* < 0.01) and lower IL-5 and MCP-1 concentrations (IL-5: 21.59 ± 24.8 (1.58-223.6) pg/ml vs 59.27 ± 38.65 (18.5-104.6) pg/ml, *p* < 0.01) MCP-1: 1351 ± 531.3 (918.0-2622) pg/ml vs 2086 ± 1269 (1667-3343) pg/ml, *p* < 0.001) (Figures 7–9).

No significant differences in IL-8, IP-10, FGF, GM-CSF, and PDGF-BB concentrations were found in any of the samples.

### 3.2. Discussion

The results of this study show significant changes in the concentrations of chemokines and GFs in the incompetent GSV and in the general circulation of CVD patients.

In the nonstimulated samples, higher concentrations of MIP-1A and MIP-1B and a lower VEGF concentration were revealed in the CVD group.

MIP-1 family is responsible for recruiting proinflammatory cells. It also plays a crucial role in the T-cell transendothelial migration [35]. Higher MIP-1A and MIP-1B concentrations in CVD patients were found in a large cytokine profile performed by Tisato et al. [36]. The elevated levels of these factors substantiate the theory of the proinflammatory impact of the turbulent blood flow in incompetent veins.

The concentrations of VEGF, a proangiogenic cytokine which promotes neovascularisation and increases vascular permeability, were found to be lower in the CVD group. Similar observations were made in a study comparing cytokine concentrations before and after endovenous laser ablation, where VEGF was found in lower concentrations in the blood of the patients before surgery [37]. In other studies, however, the concentrations of VEGF were higher in CVD patients: both in the venous tissue [22] and in the peripheral blood [36, 38]. Further studies including larger groups of patients are required to interpret the role of this factor in venous insufficiency.

Local elevation of proinflammatory markers in GSV was described by Poredos et al. [16] and in our earlier work [17]. Turbulent blood flow, venous stasis, and hypertension are well-known factors in CVD pathogenesis. Hemodynamic changes lead to the activation, adhesion, and migration of leukocytes through the venous wall, a so-called “leukocyte trap” [39]. The activated leukocytes damage the endothelium, causing inflammatory response from the endothelial cells [8, 32]. Local increase of inflammatory response is therefore postulated as an important factor of CVD pathogenesis.

In this study, eotaxin and G-CSF had higher concentrations in the incompetent saphenofemoral junction when compared with the same patients' general circulation. G-CSF stimulates neutrophil production and mobilization, attracting neutrophils to the inflammation site and restricting their activity in noninflamed regions [40]. Its higher concentration found in the incompetent vein seems to further confirm the proinflammatory effect of nonlaminar blood flow.

Eotaxin is a potent eosinophil chemoattractant, and it has a role in multiple inflammatory diseases such as asthma, atopic dermatitis, or inflammatory bowel disease [41]. It is supposed to have a local impact on tissues in atherosclerosis [41]. In this study, elevated eotaxin concentrations were found in the incompetent vein, while in a study by Sachdev et al., eotaxin was found in lower concentrations in the general circulation of varicose patients [42]. This might indicate its regional role in the pathogenesis of the disease.

Addition of PHA to the cultures revealed some differences in lymphocyte reaction to stimulation. G-CSF concentration was higher in the GSV when compared with the upper limb of the patients, just as it was in the nonstimulated samples. When stimulated samples were compared between the groups, eotaxin levels were higher in the CVD group and IL-5 and MCP-1 concentrations were lower when compared with controls. The lymphocytes in the control group produced significantly more MCP-1 in reaction to PHA than in the CVD group. No differences in VEGF concentrations were found between the groups. Very few studies discuss the role of the above factors in the CVD. In a study of chronic venous ulcer wounds, the MCP-1 concentration was elevated in the wound tissue and in the healing process, its concentrations increased [43]. In a study comparing cytokine concentrations in general circulation between a healthy group and CVD patients, MCP had lower concentrations in the CVD group [41]. A study comparing cytokine concentrations before and after surgical flow correction (so-called CHIVA procedure) showed significantly higher MCP-1 concentrations after the surgery [12]. MCP-1 is produced by a multitude of cells and acts not only as a chemotactic agent but also as an angiogenesis promotor [44, 45]. All the above results indicate the importance of MCP-1 in tissue repair [12]. Its lower concentration in the incompetent veins suggests its impact on impaired tissue healing in CVD. However, a study by Tisato et al. showed higher MCP-1 concentrations in CVD group when compared to controls [36]; therefore, more studies would be required to determine the role of these cytokines.

The increased concentration of eotaxin in the stimulated samples of CVD patients supports the hypothesis of the important role of inflammation in this disease. However, in another study, eotaxin was decreased along with other cytokines in varicose patients. The authors of the study concluded that a generally less varied inflammatory network seems to be present in CVD patients [42]. In our study, apart from VEGF (lower concentrations in the CVD group in the nonstimulated samples), IL-5 was present in significantly lower concentrations in the CVD group in the stimulated samples. This interleukin affects mainly eosinophils, basophils, and mast cells, and it is widely examined as a target in hypereosinophilic conditions [46, 47].

Other cytokines analyzed in this study were IL-8, IP-10, FGF, GM-CSF, and PDGF-BB and they did not show any significant differences in concentrations between samples. Contradictory results concerning PDGF-BB concentration in incompetent veins have been published [13, 42]. The aforementioned study assessing the effect of CHIVA on cytokine concentrations described a decrease in IP-10 and its increase after surgical flow correction [12]. Elevated concentrations of GM-CSF have also been noted [36].

In this study, only eotaxin and G-CSF showed significantly higher concentrations locally in the incompetent saphenofemoral junction in comparison with the cubital vein. This suggests that the turbulent flow may have a stimulating impact on the production of these cytokines by lymphocytes in CVD. However, other chemokines and GFs did not show any significant local concentration changes. Samples derived from the calf varices would have been exposed to more stasis and therefore other local changes in the concentrations of chemokines and GFs could have been revealed. However, blood would inevitably come from different tributaries in each patient and therefore we found it less comparable. Drawing the blood from the calf region of the great saphenous vein would also result in less comparable samples as the GSV is not exposed at the same level in all patients. The choice of saphenofemoral junction assured that the samples were obtained from the same anatomical region with most evident oscillatory flow.

Another limitation of this study is that no samples were obtained from the lower limb veins of healthy subjects. Taking blood samples from both the upper and lower limbs of healthy volunteers would expose them to too much distress and therefore has not been suggested. Some researchers have used samples from GSV grafts from patients undergoing cardiac bypass surgery as controls [6]; however, we considered such a group of patients most probably subject to numerous factors altering their immunological state (e.g., atherosclerosis, acetylsalicylic acid intake) and therefore not suitable for this study.

The potential of the lymphocytes in the incompetent veins to respond to activating factors was tested by addition of PHA to the cultures. PHA is a lymphocyte T stimulant. Therefore, the lymphocyte B response to stimulation was not assessed and requires further study.

The low number of patients is definitely another limitation of this study. The same problem was also met by other authors working on a similar subject [8, 12, 42, 48]. The unanimous results of the studies concerning cytokines in CVD require further investigation with larger groups of patients in order to determine the role of cytokines in CVD and the impact of the oscillatory flow on the functioning of immunological cells.

## 4. Conclusions

The results obtained in this study show that CVD lymphocytes produce cytokines responsible for recruiting inflammatory cells, angiogenesis, and tissue healing in significantly different concentrations in comparison with a healthy group. The differences are also present when GSV samples are compared with the patients' general circulation. This supports the theory that the turbulent flow present in the incompetent veins affects the functioning of the immunological cells, which may have an important impact on the pathogenesis of the disease. The exact nature of these changes requires further investigation in larger groups of patients.

## Figures and Tables

**Table 1:** Comparison of the cytokine concentrations between samples with PHA-stimulated and unstimulated lymphocytes.

Cytokine	CVD group		Control group—upper limb
	Lower limb	Upper limb	
Eotaxin	*p* < 0.001	*p* < 0.001	*p* < 0.05
IL-8	*p* < 0.05	*p* < 0.05	NS
MIP-1A	*p* < 0.001	*p* < 0.001	*p* < 0.05
MIP-1B	NS	NS	NS
IP-10	*p* < 0.000001	*p* < 0.000001	*p* < 0.05
MCP-1	*p* < 0.00001	*p* < 0.0001	*p* < 0.05
IL-5	NS	NS	*p* < 0.05
FGF	NS	*p* < 0.05	*p* < 0.05
G-CSF	*p* < 0.000001	*p* < 0.000001	*p* < 0.05
GM-CSF	NS	*p* < 0.05	NS
PDGF-BB	NS	NS	NS
VEGF	*p* < 0.05	*p* < 0.0001	NS

Statistically significant increase of concentration in PHA-stimulated samples: *p* ≤ 0.05. NS: no significant change in cytokine concentration, *p* > 0.05.

## Data Availability

The Bio-Plex data used to support the findings of this study are available from the corresponding author upon request.
